# Acknowledgement of manuscript reviewers 2014

**DOI:** 10.1186/s40201-015-0163-5

**Published:** 2015-03-10

**Authors:** Simin Nasseri, Amir Hossein Mahvi, Mitra Gholami, Nematallah Jaafarzadeh, Mohammad Reza Monazzam, Ramin Nabizadeh Nodehi, Kazem Naddafi, Noushin Rastkari, Roshanak Rezaei, Kamyar Yaghmaeian, Masoud Yunesian

**Affiliations:** Tehran University of Medical Sciences, Tehran, Iran; School of Public Health, Tehran University of Medical Sciences, Tehran, Iran; Ahvaz Jondishapur University of Medical Sciences, Ahvaz, Iran

## Abstract

The editors of *Journal of Environmental Health Science & Engineering* would like to thank all our reviewers who have contributed to the journal in Volume 12 (2014).

## Reviewer acknowledgement

**Nasser Abdel-Latif**

Egypt

**Abdul Halim Abdullah**

Malaysia

**Rosa Abellana**

Spain

**Mehrnoosh Abtahi**

Iran

**Ebrahim Afshari**

Iran

**Pooyaneh Aghapoorkhameneh**

Iran

**Reza Ahmadkhaniha**

Iran

**Farhang Akbar-Khanzadeh**

United States Of America

**Mohsen Aliabadi**

Iran

**Mahmood Alimohammadi**

Iran

**Basim Almayahi**

Malaysia

**Hassan Amini**

Iran

**Mehwish Anis**

Pakistan

**Mohsen Arbabi**

Iran

**Zahra Asadgol**

Iran

**Hosseinali Asgharnia**

Iran

**Hasan Aslani**

Iran

**Mohammad Baghapour**

Iran

**Mohammad Mehdi Baneshi**

Iran

**Edris Bazrafshan**

Iran

**S. Mehdi Borghei**

Iran

**Justin Brooks**

Australia

**Joanna Burger**

United States Of America

**Alireza Chackoshian Khorasani**

Iran

**Shri Chand**

India

**Yousef Dadban Shahamat**

Iran

**Hadi Daneshmandi**

Iran

**Carla De Carvalho**

Portugal

**Dirk De Korte**

Netherlands

**Mansooreh Dehghani**

Iran

**Emad Dehghanifard**

Iran

**Bhatkhande Dhananjay**

India

**Sina Dobaradaran**

Iran

**Claudia Domini**

Argentina

**Mansour Ebrahimi**

Iran

**Mojtaba Ehsanifar**

Iran

**Chris Ekpenyong**

Nigeria

**Akbar Eslami**

Iran

**M.A. Faramarzi**

Iran

**Mehrdad Farrokhi**

Iran

**Mehdi Fazlzadeh**

Iran

**Roberto Fernandez-Lafuente**

Spain

**Guadalupe García-Elorriaga**

Mexico

**Merlina Georges**

France

**Mehrorang Ghaedi**

Iran

**Vahideh Ghaffarian**

Iran

**Farshid Ghanbari**

Iran

**Mohammad Ghanbari Ghozikali**

Iran

**Mohammad Taghi Ghaneian**

Iran

**Zahra Ghobadi Nejad**

Iran

**Mitra Gholami**

Iran

**Fathhollah Gholami Borujeni**

Iran

**Akbar Gholampour**

Iran

**Abdolmajid Gholizadeh**

Iran

**Stefanos Giannakis**

Switzerland

**Saeid Gitipour**

Iran

**Hatam Godini**

Iran

**Gholamreza Goudarzi**

Iran

**Sanjay Govindwar**

India

**Mark Green**

United States Of America

**Yaghoub Hajizadeh**

Iran

**Nidhi Hans**

United States Of America

**Yalda Hashempour**

Iran

**Wail Hayajneh**

Jordan

**Sadegh Hazrati**

Iran

**Mohammad Hoseini**

Iran

**Gholam Ali Hoshyaripour**

Germany

**Ghulam Hussain**

Pakistan

**Muataz Hussien**

Jordan

**Syazwani Idrus**

Malaysia

**Ali Jafari**

Iran

**Mehran Javanbakht**

Iran

**Mehjbeen Javed**

India

**Allahbakhsh Javid**

Iran

**Ahmad Jonidi**

Iran

**Park Joo-Yang**

Korea, South

**Sahand Jorfi**

Iran

**Chihju George Jou**

Taiwan

**Babak Kakavandi**

Iran

**Maria Kanellaki**

Greece

**Kurunthachalam Kannan**

United States Of America

**Hussein Kaoud**

Egypt

**Êmine Erman Kara**

Turkey

**Anna Karakatsani**

Greece

**Behrooze Karimi**

Iran

**Hamid Karyab**

Iran

**Homa Kashani**

Iran

**Athanasios Katsoyiannis**

United Kingdom

**Kazem Khalagi**

Iran

**Hassan Khorsandi**

Iran

**Maryam Kiani Sadr**

Iran

**Oyeshola Kofoworola**

Thailand

**Fariba Koohdani**

Iran

**David Kreutzweiser**

Canada

**Anoop Krishnan Krishnan**

India

**Bulgariu Laura**

Romania

**Mostafa Leili**

Iran

**Luc Letenneur**

France

**Robert Levy**

United States Of America

**Jennifer Long**

Australia

**Samaneh M. B. Fard**

Australia

**Kenneth Mackenzie**

New Zealand

**Mitra Mahdavi-Mazdeh**

Iran

**Niyaz Mohammad Mahmoodi**

Iran

**Amir Hossein Mahvi**

Iran

**Fereshteh Majlessi**

Iran

**Abdelhadi Makan**

Morocco

**Afshin Maleki**

Iran

**Nabiollah Mansouri**

Iran

**Jamson Masih**

India

**David Massey**

India

**Mohammad Reza Mehrasbi**

Iran

**Mohammad Reza Mehrnia**

Iran

**Izabela Michalak**

Poland

**Veniamin Mikryakov**

Russian Federation

**Ghorbanali Mohammadi**

Iran

**Aliakbar Mohammadi**

Iran

**Mehran Mohammadian Fazli**

Iran

**Fariba Mohsenzadeh**

Iran

**Nader Mokhtarani**

Iran

**Mehdi Mokhtari**

Iran

**S. Ahmad Mokhtari**

Iran

**Ebrahim Molaee Aghaee** Iran

**Elsayed Mostafa Elsayed**

Egypt

**Ahmet Edip Müftüoglu**

Turkey

**Shafiq Mujahid**

Pakistan

**Mahdiyeh Naderzadeh**

Iran

**Mahabir Nain**

India

**Indu Nambi**

India

**Jacek Namiesnik**

Poland

**Simin Nasseri**

Iran

**Parvin Nassiri**

Iran

**Shahrokh Nazmara**

Iran

**Evangelia Nena**

Greece

**Mojtaba Noury**

Iran

**H. Kurtulus Ozcan**

Turkey

**Saeedreza Pakzad**

Iran

**Abbas Parham**

Iran

**Daniel Peptenatu**

Romania

**Giuliano Premier**

United Kingdom

**Mohammad Rafiee**

Iran

**Mohammad Reza Rahimpour**

Iran

**Bahman Ramavandi**

Iran

**Hamid Rashedi**

Iran

**Noushin Rastkari**

Iran

**Reza Rezaee**

Iran

**Roshanak Rezaei Kalantary**

Iran

**Mohammadreza Rouini**

Iran

**Imanol Ruiz-Zarzuela**

Spain

**Gasaymeh S**

Jordan

**Gholamhossein Safari**

Iran

**Ali Safarivaryani**

Iran

**Mona Salimi**

Iran

**Sadegh Samadi**

Netherlands

**Mohammad Taghi Samadi**

Iran

**Petros Samaras**

Greece

**Diana Sannino**

Italy

**Abbas Shahsavani**

Iran

**Seyed J Shahtaheri**

Iran

**Mansour Shamsipour**

Iran

**Ashkan Shokri**

Iran

**Nikhat Siddiqi**

Saudi Arabia

**Susana Silva Martinez**

Mexico

**Dipika Singh**

United States Of America

**Rajeev Pratap Singh**

Malaysia

**Patricia Siques**

Chile

**Mansooreh Soleimani**

Iran

**Bubak Souri**

Iran

**Mohammad Hossein Sowlat**

Iran

**Mustafa Soylak**

Turkey

**Delia Teresa Sponza**

Turkey

**Emanoil Surducan**

Romania

**Hassan Taghipour**

Iran

**Ajay Taneja**

India

**Pari Teymouri**

Iran

**Kalyan Raman V**

India

**Alireza Vejdani Noghreiyan**

Iran

**Jun Wang**

China

**Rasoul Yarahmadi**

Iran

**Mihriban Yilmaz Civan**

Turkey

**Chiu-Chung Young**

Taiwan

**Masud Yunesian**

Iran

**Narges Zaredar**

Iran

**Mahdi Zargham**i

United States Of America

**Mohammad Ali Zazouli**

Iran

**Murat Zengin**

Turkey

**Baowei Zhao**

China

**Ping Zhuang**

China

